# Patients Hospitalized for COVID-19 in the Periods of Delta and Omicron Variant Dominance in Greece: Determinants of Severity and Mortality

**DOI:** 10.3390/jcm12185904

**Published:** 2023-09-11

**Authors:** Vagia Karageorgou, Andriana I. Papaioannou, Maria Kallieri, Myrto Blizou, Stefanos Lampadakis, Maria Sfika, Antonios Krouskos, Vasileios Papavasileiou, Franceska Strakosha, Kalliopi Theoni Vandorou, Pavlos Siozos, Marina Moustaka Christodoulou, Georgia Kontonasiou, Vasiliki Apollonatou, Elvira Markella Antonogiannaki, Christos Kyriakopoulos, Christina Aggelopoulou, Christos Chronis, Konstantinos Kostikas, Evangelia Koukaki, Zoi Sotiropoulou, Athanasia Athanasopoulou, Petros Bakakos, Pinelopi Schoini, Emmanouil Alevrakis, Sotirios Poupos, Evangelia Chondrou, Dionisios Tsoukalas, Alexia Chronaiou, George Tsoukalas, Sofia Koukidou, Georgios Hillas, Katerina Dimakou, Konstantinos Roukas, Ifigeneia Nakou, Diamantis Chloros, Evangelia Fouka, Spyros A. Papiris, Stelios Loukides

**Affiliations:** 1 2nd Respiratory Medicine Department, “Attikon” University Hospital, Athens Medical School, National and Kapodistrian University of Athens, 12462 Athens, Greece; vagiakar@hotmail.com (V.K.); kallierimaria@yahoo.gr (M.K.); myrto_bl@hotmail.com (M.B.); steflamp17@gmail.com (S.L.); maria.sfka@gmail.com (M.S.); strakosia.eirini@gmail.com (F.S.); vicky_apoll@hotmail.com (V.A.);; 2 1st Respiratory Medicine Department, “Sotiria” Chest Hospital, Athens Medical School, National and Kapodistrian University of Athens, 11527 Athens, Greece; papaioannouandriana@gmail.com (A.I.P.); zoisotiropoulou96@gmail.com (Z.S.); petros44@hotmail.com (P.B.); 3 4th Respiratory Medicine Department, “Sotiria” Chest Hospital, 11527 Athens, Greece; kantonogiannaki@gmail.com (E.M.A.); pinelopi_schoini@hotmail.com (P.S.); m.alevrakis@gmail.com (E.A.); pouposs@hotmail.com (S.P.); elinachondrou@yahoo.gr (E.C.); chronaiou@yahoo.gr (A.C.);; 4 Respiratory Medicine Department, University Hospital of Ioannina, 45500 Ioannina, Greece; ckyriako@yahoo.gr (C.K.); krapsi@hotmail.com (C.C.); ktkostikas@gmail.com (K.K.); 5 5th Respiratory Medicine Department, “Sotiria” Chest Hospital, 11527 Athens, Greece; sofiek90@hotmail.com (S.K.); ghillas70@yahoo.gr (G.H.); kdimakou@yahoo.com (K.D.); 6 COVID-19 Clinic, General Hospital G. Papanikolaou, Aristotle University of Thessaloniki, 57010 Thessaloniki, Greeceifigenianakou@gmail.com (I.N.); dchloros@msn.com (D.C.); evafouka@gmail.com (E.F.)

**Keywords:** COVID-19, SARS-CoV-2, variant, mortality, Omicron, Delta

## Abstract

Background: Coronavirus disease 2019 (COVID-19) has been a pandemic since 2020, and depending on the SARS-CoV-2 mutation, different pandemic waves have been observed. The aim of this study was to compare the baseline characteristics of patients in two phases of the pandemic and evaluate possible predictors of mortality. Methods: This is a retrospective multicenter observational study that included patients with COVID-19 in 4 different centers in Greece. Patients were divided into two groups depending on the period during which they were infected during the Delta and Omicron variant predominance. Results: A total of 979 patients (433 Delta, 546 Omicron) were included in the study (median age 67 years (54, 81); 452 [46.2%] female). Compared to the Omicron period, the patients during the Delta period were younger (median age [IQR] 65 [51, 77] vs. 70 [55, 83] years, *p* < 0.001) and required a longer duration of hospitalization (8 [6, 13] vs. 7 [5, 12] days, *p* = 0.001), had higher procalcitonin levels (ng/mL): 0.08 [0.05, 0.17] vs. 0.06 [0.02, 0.16], *p* = 0.005, ferritin levels (ng/mL): 301 [159, 644] vs. 239 [128, 473], *p* = 0.002, C- reactive protein levels (mg/L): 40.4 [16.7, 98.5] vs. 31.8 [11.9, 81.7], *p* = 0.003, and lactate dehydrogenase levels (U/L): 277 [221, 375] vs. 255 [205, 329], *p* < 0.001. The Charlson Comorbidity Index was lower (3 [0, 5] vs. 4 [1, 6], *p* < 0.001), and the extent of disease on computed tomography (CT) was greater during the Delta wave (*p* < 0.001). No evidence of a difference in risk of death or admission to the intensive care unit was found between the two groups. Age, cardiovascular events, acute kidney injury during hospitalization, extent of disease on chest CT, D-dimer, and neutrophil/lymphocyte ratio values were identified as independent predictors of mortality for patients in the Delta period. Cardiovascular events and acute liver injury during hospitalization and the PaO_2_/FiO_2_ ratio on admission were identified as independent predictors of mortality for patients in the Omicron period. Conclusions: In the Omicron wave, patients were older with a higher number of comorbidities, but patients with the Delta variant had more severe disease and a longer duration of hospitalization.

## 1. Introduction 

Coronavirus disease 2019 (COVID-19) was declared a global pandemic by the World Health Organization (WHO) on 11 March 2020 [1]. The disease is caused by the severe acute respiratory syndrome coronavirus 2 (SARS-CoV-2), and the first cases were reported in Wuhan, China, in late December 2019 [2]. Since being declared a global pandemic, COVID-19 has rapidly spread around the world and has become a worldwide health threat [3], with more than 767 million cases and more than 6.9 million deaths [4].

Like other RNA viruses, SARS-CoV-2 evolves as changes in the genetic code occur, leading to the emergence of multiple variants depending on the mutation of the virus [5,6]. The so-called Delta variant (B.1.617.2) was first detected in October 2020 in India and has expanded rapidly, dominating almost every country by the end of 2021 [7]. However, in November 2021, a new variant of concern, called Omicron (B.1.1.529), was identified in South Africa. Omicron includes a large number of mutations, is more transmissible with higher viral binding affinity, and has rapidly supplanted the Delta variant [8,9]. 

The end of the pandemic left us wiser regarding the risk factors associated with severe COVID-19 infection, showing that older age, multiple and/or significant comorbidities, and the absence of immunization against SARS-CoV-2 are all predictors of more severe disease and an unfavorable outcome [10,11,12]. However, despite a higher vaccination status among patients infected with the Omicron variant compared to the Delta variant, there is evidence that these two different COVID-19 subtypes differ regarding their ability to cause severe disease [13,14]. 

The aim of the present study was to compare the characteristics of the patients hospitalized for severe COVID-19 during the periods of the pandemic with the Delta and Omicron predominances, to compare indicators of disease severity caused by the two different variants, and to examine the possible predictors of mortality during these two different periods. 

## 2. Materials and Methods

### 2.1. Study Design 

This observational retrospective analysis was conducted in 6 COVID-19 clinics in 4 different hospitals in Greece, including the 2nd Respiratory Medicine Department, “Attikon” University Hospital, the National and Kapodistrian University of Athens, the 1st Respiratory Medicine Department, “Sotiria” Chest Hospital, the National and Kapodistrian University of Athens, the 4th Respiratory Medicine Department, “Sotiria” Chest Hospital, Athens, the Respiratory Medicine Department, University Hospital of Ioannina, the 5th Respiratory Medicine Department, “Sotiria” Chest Hospital, Athens, and the COVID-19 Clinic, General Hospital G. Papanikolaou, Aristotle University of Thessaloniki. All patients hospitalized with COVID-19 were included in the study and were divided into 2 groups according to the time of hospital admission. The first group included admissions from 13 November 2021 to 15 January 2022 while the second group included admissions after 16 of January 2022 to 8 June 2022, corresponding to the dominance of the Delta and Omicron variants, respectively, according to local regulation of epidemiological surveillance (https://eody.gov.gr/, accessed on 25 June 2023). Inclusion criteria were documented SARS-CoV-2 infection and either severe disease requiring inpatient treatment or significant comorbidities that resulted in the patient being characterized as high risk for COVID-19 complications. The diagnosis of COVID-19 infection was based on a positive nasopharyngeal swab for SARS-CoV-2 using the polymerase chain reaction (PCR) methodology [15]. 

Epidemiological characteristics (age, gender, body mass index (BMI), smoking history), comorbidities and Charlson Comorbidity Index (CCI) score, symptoms on admission to the hospital, vaccination status, laboratory tests (white blood cells, lymphocytes, neutrophil to lymphocyte ratio (NLR), platelets, D-dimer, procalcitonin (PCT), C-reactive protein (CRP), lactate dehydrogenase (LDH), ferritin), oxygen requirement, extent of disease according to the computed tomography (CT) imaging results (percentage of pulmonary involvement), intensive care unit (ICU) admission, events during hospitalization (cardiovascular event, acute kidney and liver injury) were collected, as well as the outcome data. The cardiovascular events included diseases of the heart, vascular diseases of the brain, and diseases of blood vessels, as defined by WHO [16]. 

### 2.2. Characterization of Vaccination Status

Fully vaccinated adults were considered those who had received at least two doses of an mRNA vaccine during the Delta period (or one dose of the Johnson vaccine) (since the booster dose was not recommended during that period of time) and those who had received at least three vaccine doses (or at least one booster dose if the initial dose was with the Johnson vaccine) during the Omicron period. Unvaccinated subjects were considered those who did not receive any vaccine dose, while partially vaccinated subjects were those who had received 1 dose during the Delta period and 1 or 2 doses of the vaccine during the Omicron period.

### 2.3. Imaging Results

The extent of disease according to computed tomography (CT) findings was estimated according to the COVID-19 visual assessment scale (Co.V.A.Sc), which is a visual assessment scale that roughly estimates the percentage of pulmonary parenchyma affected by COVID-19, as seen on chest CT, when both lungs are evaluated as a whole (0%, 1–10%, 11–25%, 26–50%, 51–75%, and >75%) [17]. 

### 2.4. Assessment of Comorbidities

The presence of comorbidities was recorded according to the patients’ medical files and the Charlson Comorbidity Index (CCI) [18], which is known to evaluate a vast number of comorbidities and was calculated on admission. Higher Charlson Comorbidity Index (CCI) scores are known to be predictors of increased mortality [18,19,20]. Cardiovascular disease includes hypertension, coronary artery disease, heart failure, myocardial infarction, atrial fibrillation, and vascular disease. For the characterization of specific acute diseases appearing as complications of severe COVID-19 infection during hospitalization, specific guidelines were used, i.e., acute kidney injury was diagnosed according to the KDIGO clinical practice guidelines [21] and acute liver injury as defined by David G. et al. and the Acute Liver Failure Study Group [22]. COVID-19 contributes to cardiovascular events during hospitalization, including acute myocardial injury as a result of acute coronary syndrome, myocarditis, stress cardiomyopathy, arrhythmias, cardiogenic shock, and cardiac arrest [23,24].

## 3. Statistical Analysis

Patients’ characteristics were described for the whole population and according to the SARS-CoV-2 variant (Delta or Omicron). The normality of distributions was checked using the Kolmogorov–Smirnov test. Categorical data were summarized using frequency counts and percentages. Continuous data were expressed as mean plus standard deviation (SD) or median and interquartile range (IQR; 25th and 75th percentile), for normal and skewed distributions, respectively. Standard tests were used to check univariate associations between categorical and categorical (Fisher’s exact tests or chi-squared tests) or categorical and continuous variables (Mann–Whitney test). *p*-values < 0.05 indicated statistical significance. Cox regression analysis, univariate and multivariate, was used to evaluate possible predictors of mortality. The determinants were set as independent predictors, and odds ratios (OR) with 95% confidence intervals (95% CI) were estimated. Statistical analysis was performed using the SPSS 23 statistical package (SPSS Inc., Chicago, IL, USA).

## 4. Results

### 4.1. Characteristics of the Study Participants 

A total of 979 patients hospitalized for COVID-19 were included in the study; 452 (46.2%) were women and 527 (53.8%) were men, and the median age was 67 years (IQR, 54 to 81 years). The Delta-predominant period of the pandemic included 433 (44.2%) patients, while the Omicron period included 546 (55.8%) patients. Compared to the Omicron period, the median age (years) of patients during the Delta period was significantly lower {median age [IQR] 65 [51, 77] vs. 70 [55, 83], *p* < 0.001}. Interestingly, in the total sample of study subjects, 519 (53%) patients had never smoked, and 145 (14.8%) were obese. The demographic data of all patients included in the study are represented in Table 1.

### 4.2. Comorbidities in Patients with COVID-19 Hospitalized during the Two Waves of the Pandemic

Regardless of the pandemic period, the prevalent comorbidity was cardiovascular disease, found in 572 (58.4%) patients, followed by diabetes mellitus in 206 (21%). Chronic obstructive pulmonary disease (COPD) and asthma as comorbidities were recorded in a minority of patients with a frequency of 10.1% (n = 99) and 6% (n = 59), respectively. The Charlson Comorbidity Index was significantly higher during the Omicron wave compared to the Delta wave, {median [IQR] 4 [1, 6] vs. 3 [0, 5], *p* < 0.001}. Regarding comorbidities, there was a statistically significant difference between periods of Omicron and Delta predominance in the frequency of patients with cardiovascular disease (62.1% vs. 53.8%, *p* = 0.009), connective tissue disease (4.9% vs. 2.3%, *p* = 0.032), dementia (16.8% vs. 10.6%, *p* = 0.005), idiopathic pulmonary fibrosis (2% vs. 0.5%, *p* = 0.035), chronic kidney disease (6.6% vs. 3.7%, *p* = 0.045), and chronic treatment of immunosuppressive drugs (9.9% vs. 5.1%, *p* = 0.005), respectively (Table 2). 

### 4.3. Symptoms on Admission during the Two Study Periods 

Regarding symptoms at admission to the hospital, dyspnea (45.9%), cough (41%), and fever (39%) were the predominant ones. The main symptoms did not differ between the two study groups; however, the patients during the period of Omicron prevalence had more frequently gastrointestinal symptoms, such as anorexia, nausea, vomiting, and abdominal pain, compared to patients during the period of Delta predominance (20.5% vs. 14.3%, respectively, *p* = 0.012), while patients during the period of Delta prevalence more frequently reported fever compared to the patients of the period with Omicron predominance (44.6% vs. 34.6%, respectively, *p* = 0.002) (Table 3). 

### 4.4. Laboratory Findings 

Among baseline laboratory parameters, significantly higher median white blood cell counts were recorded in patients hospitalized during the Omicron wave, while higher median procalcitonin, ferritin levels, serum LDH, and CRP levels were documented more frequently in the period of Delta variant dominance (Table 3).

### 4.5. Disease Severity and Outcomes during the Two Waves of the Pandemic

During the period where the Omicron was the predominant variant, 85 patients (15.6%) had a fatal outcome, while 54 deaths (12.5%) occurred among our study subjects during the Delta wave. There was no statistically significant difference in ICU admissions between the two groups (6.5% vs. 5.1%, *p* = 0.37). Regarding disease severity, the PaO_2_/FiO_2_ ratio on admission during the Delta period was lower [314 (257, 360) vs. 322 (269, 368), *p* = 0.053], compared to the period with the Omicron predominance, although the difference was not significant, while the duration of hospitalization was longer [8 (6, 13) vs. 7 (5, 12) days, *p* = 0.001] and the extent of the COVID-19-associated pulmonary opacities according to the findings on chest CT (available in a subgroup of 606 patients) was greater (*p* < 0.001). However, no differences in maximum FiO_2_ requirements were observed between the two groups (Table 4).

COVID-19 treatment was according to national protocols. In total, 741 (75.7%) of patients received dexamethasone, 661 (67.5%) received remdesivir, 37 (3.8%) received baricitinib, 39 (4%) received tocilizumab, and 10 (1%) received anakinra. Significantly more patients were treated with both dexamethasone (79.0% vs. 73.1%, *p* = 0.036) and remdesivir (70.9% vs. 64.8%, *p* = 0.044) during the Delta wave compared to the Omicron wave, whereas there was no significant difference in the two waves regarding the administration of baricitinib (4.4% vs. 3.3%, *p* = 0.374), tocilizumab (4.2% vs. 3.8%, *p* = 0.805), and anakinra (0.7% vs. 1.3%, *p* = 0.362). 

### 4.6. Complications during Hospitalization for COVID-19 in the Two Study Periods

During hospitalization, patients of the Omicron predominant period experienced more cardiovascular events, deep vein thrombosis (DVT), and acute liver injury (10.4% vs. 3.5%, *p* < 0.001), 1.8% vs. 0%, *p* = 0.005, and 11.2% vs. 6.9%, *p* = 0.023 for the Omicron and the Delta periods, respectively. Furthermore, there was also a trend for more acute kidney injury in the period of Omicron compared to the period of Delta predominance, although this difference did not reach statistical significance (14.8% vs. 10.6%, *p* = 0.051) (Table 5).

### 4.7. Vaccination Status during the Two Waves of the Pandemic

**Figure 1:**
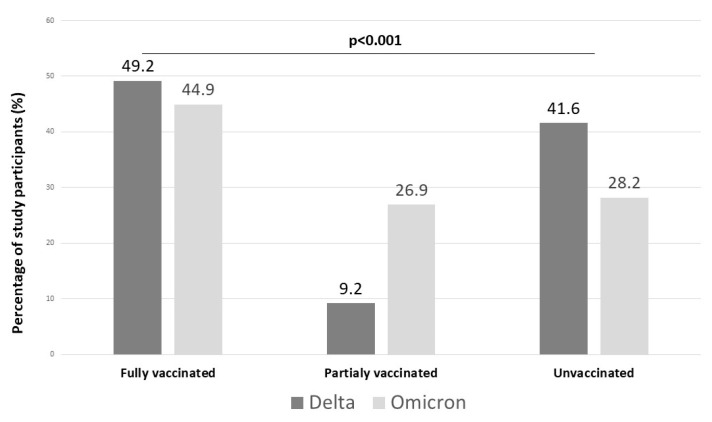
Vaccination status of hospitalized patients during Delta and Omicron predominant periods in Greece.

During the period of Delta variant predominance, 213 (49.2%) of patients were fully vaccinated, 40 (9.2%) were partially vaccinated, and 180 (41.6%) were unvaccinated. During the period of Omicron predominance, 245 (44.9%) were fully vaccinated, 147 (26.9%) were partially vaccinated, and 154 (28.2%) were unvaccinated. The vaccination status of the study subjects during the two waves of the pandemic is shown in Figure 1.

In the multivariate Cox regression analysis during the Delta wave, the age of patients, the neutrophil to lymphocyte ratio (NLR), the D-dimer value on admission, the appearance of an acute cardiovascular event or acute kidney injury during hospitalization, and the extent of disease on the CT scan were independent predictors of mortality. During the Omicron variant dominance, the only independent predictors of mortality were the appearance of an acute cardiovascular event or acute liver injury during hospitalization and the level of PaO_2_/FiO_2_ ratio on admission. The univariate and multivariate regression analyses for each period of the pandemic are shown in Table 6A,B. 

## 5. Discussion

In this retrospective observational study with 979 Greek patients admitted to the hospital during the two periods of the pandemic caused by the Delta and Omicron variants, we have shown differences regarding the age of hospitalized patients and the frequency of several comorbidities, as well as the total comorbidity burden of the patients according to the Charlson Comorbidity Index. Patients hospitalized during the Delta wave expressed higher levels of procalcitonin, ferritin, CRP, and LDH on admission compared to those admitted during the Omicron wave. Interestingly, although patients experienced similar symptoms during both periods, fever appeared more frequently during the Delta period, while gastrointestinal symptoms appeared more frequently during the Omicron period. The duration of hospitalization was longer during the Delta wave, while the extent of disease, according to the CT findings, was also greater during this period. On the contrary, during the Omicron wave, adverse events related to the cardiovascular system and liver and events of deep vein thrombosis appeared more frequently. Finally, age, D-dimer value, neutrophil to lymphocyte ratio, cardiovascular events and acute kidney injury during hospital stay, and the extent of disease on chest CT were independent predictors of mortality during the Delta period, while during the Omicron period, independent predictors of mortality were cardiovascular events and acute liver injury during hospital stay and the PaO_2_/FiO_2_ ratio on admission.

Patients hospitalized for COVID-19 during the Omicron wave were older and suffered more frequently from significant comorbidities compared to patients hospitalized during the Delta wave. This is in accordance with preview studies conducted in the United States [13,25] showing that during the Omicron wave, hospitalized patients for COVID-19 were older and had more impaired health status, leading to the possible conclusion that the two variants of SARS-CoV-2 differ in their pathogenesis. The fact that in our study the percentage of patients who were totally vaccinated did not differ significantly probably indicates that the Omicron variant causes severe disease mostly in patients with impaired health status [26], while the Delta variant could lead to severe disease in young patients without significant comorbidities. This assumption is also in accordance with preview studies, which report that Omicron infection has reduced overall severity compared with Delta [27,28,29,30]. The fact that the duration of hospitalization was greater during the Delta wave also supports this hypothesis.

In our study, patients hospitalized during the period of Delta variant predominance had a greater extent of disease according to computed tomography, and it is known that the higher the quantitative burden of pulmonary parenchyma affected by COVID-19, the more severe the disease [31,32]. Furthermore, the patients hospitalized for COVID-19 during the Delta wave required a longer duration of hospitalization, which is in line with other studies [33]. Additionally, they presented higher levels of inflammatory parameters such as PCT, ferritin, CRP, and LDH levels, and these observations are also consistent with previous studies [34,35,36], showing that the inflammatory response of infected patients seems to be lower when disease is caused by the Omicron variant.

In our study, we observed that during the Omicron wave, adverse events related to the cardiovascular system and the liver and events of deep vein thrombosis appeared more frequently. This might be in accordance with the aforementioned observation that patients admitted to the hospital for severe COVID-19 during the Omicron wave suffered from more comorbidities and, in general, had a more impaired health status. This impaired health status, including age-related physiological changes, impaired immune function, and preexisting illnesses, might also be related to the increased risk of experiencing adverse events. It is plausible that COVID-19-associated coagulopathy is a downstream consequence of the host’s inflammatory response to SARS-CoV-2 and innate immune activation [37,38]. The triggering of host inflammatory reactions results in increased production of cytokines that have pleiotropic effects, including activation of coagulation and thrombin generation as critical communication components among humoral and cellular amplification pathways, a term called thromboinflammation or immunothrombosis. The term ‘thromboinflammation’ refers to a process in which inflammation and thrombosis coexist within microvessels in response to harmful stimuli [39]. The inflammatory effects of cytokines also result in activated vascular endothelial cells and endothelial injury with resultant prothrombotic properties [40]. However, it is important to mention that the appearance of cardiovascular events was an independent risk factor of mortality during both waves, whether acute kidney disease during hospital stay was an independent predictor of mortality only during the Delta wave and acute liver disease only in the Omicron wave. Systemic inflammation seems to be the most prominent mechanism underlying the cardiovascular complications of COVID-19 [41].

Regarding the symptoms the patients experienced upon admission, fever occurred more frequently during the Delta wave, while gastrointestinal events were more frequent during the Omicron wave. The increased frequency of patients reporting fever might be related to the increased inflammatory burden observed during the Delta period [34,35,36].

Our results present no statistically significant difference in mortality for the two pandemic waves. Our observation is in contrast with other studies in Canada and England [14,42], which have shown that mortality from COVID-19 during the Omicron period was lower. Our results could be explained by the fact that although the Delta variant seems to cause more severe disease, the patients during the Omicron variant predominance period were older, suffered from more comorbidities, and experienced more complications during hospitalization; therefore, they were more likely to encounter death. Furthermore, a recent study conducted in patients with severe COVID-19 admitted to the ICU has shown that older age was not associated with more severe disease or worse outcomes since the presence of comorbidities even in younger patients seemed to play the predominant role [11].

Previous studies have evaluated possible predictors of mortality for COVID-19 [10,12,43,44]. Interestingly, in our study, risk factors for mortality differed between the two waves. Age was an independent predictor of mortality during the Delta wave but not during the Omicron wave. This can be explained by the fact that patients admitted to the hospital during the Delta wave were younger, so those of older age might have decreased reserve and a more impaired general health status. Furthermore, the fact that patients during the Omicron wave were elderly could also explain the absence of age as a significant predictor of mortality in this group. The D-dimer value was a predictor of mortality during the period of Delta variant predominance but not during the Omicron wave of the pandemic, although there is a trend in the latest. This observation is in accordance with previous meta-analyses [45,46], showing that the D-dimer value measured on admission was significantly correlated with the severity of COVID-19 pneumonia and could predict mortality in hospitalized patients. The neutrophil to lymphocyte ratio was an independent predictor of mortality during the Delta period but not during the Omicron period. Previous studies have shown that NLR has good predictive value for disease severity and mortality in patients with COVID-19 [47,48]. It is known that elevated NLR is associated with dysregulated expression of several inflammatory cytokines, an increase in regular low-density neutrophils, and the upregulation of several genes that play a crucial role in the lymphocyte apoptosis pathway provoked by the pathogenetic mechanisms of SARS-CoV-2 infection [49]. The fact that, as already mentioned, the inflammatory burden is greater in patients infected during the Delta wave might also explain this observation.

The extent of disease on the chest CT was an independent predictor of mortality during the Delta period but not during the Omicron period. A previous study has shown that CT findings were associated with the patient’s outcome [50]. However, the fact that during the Omicron wave, the extent of disease on CT was not associated with mortality could lead to the hypothesis that mortality during this wave was mainly related to the total health status of the patients and not to the severity of COVID-19.

This study contains some limitations. First, there was no molecular identification of the SARS-CoV-2 variant, and we have divided patients into two groups according to the available epidemiological data in our country. However, we have included the two different timepoints at which epidemiological data showed the predominance of the Delta and Omicron variants. Accordingly, we can support that the Delta variant was responsible for causing infection in group 1 (since the omicron variant had not yet appeared during this period) and the Omicron variant in group 2 (since at this time the omicron variant was the main variant detected in the population and the sewage according to the local regulation of epidemiological surveillance (https://eody.gov.gr/, accessed on 25 June 2023). Furthermore, we only recorded the number of vaccines the patient received and not whether patients had received a possible immunization through a previous SARS-CoV-2 infection. Finally, CT findings were available only in a subgroup of 606 patients (61.9%), which might affect the results of our observations regarding the significance of disease extension on chest CT on the patients’ outcomes, while we have to admit that the COVID-19 visual assessment scale (Co.V.A.Sc) is affected by inter-operator variability and standardization of the results was not performed since CTs were interpreted routinely by the responsible radiologist of each hospital.

In conclusion, we have shown that the characteristics of patients hospitalized for COVID-19 differed between the two waves of the pandemic, with the Delta variant causing more severe disease and affecting patients with better previous health status, while the Omicron variant affected mainly older patients suffering from several comorbidities. Since the COVID-19 pandemic seems to belong in history, our observations can be valuable knowledge for future pandemics and help to recognize early patients who will probably be at increased risk for unfavorable outcomes.

## Figures and Tables

**Table 1:** Demographic characteristics of patients hospitalized during periods of Delta and Omicron predominant variants.

Characteristics			Total (n = 979)	Delta (n = 433)	Omicron (n = 546)	*p* Value
Age (median, IQR)			67 (54, 81)	65 (51, 77)	70 (55, 83)	**<0.001**
Gender (n, %)		Female	452 (46.2%)	212 (49%)	240 (44%)	0.119
		Male	527 (53.8%)	221 (51%)	306 (56%)	
Obesity (BMI > 30 kg/m^2^) (n, %)			145 (14.8%)	61 (14.1%)	84 (15.4%)	0.614
Smoking habit (n, %)	Never		519 (53%)	257 (59.4%)	262 (48%)	**0.002**
	Current		184 (18.8%)	70 (16.2%)	114 (20.9%)	
	Former		276 (28.2%)	106 (24.5%)	170 (31.1%)	

Data are presented as n (%) unless otherwise indicated. Bold indicates statistically significant differences. Abbreviations: BMI: body mass index, IQR: interquartile range, n: number.

**Table 2:** Comorbidities of patients included in the study.

Comorbidities	Total (n = 979)	Delta (n = 433)	Omicron (n = 546)	*p*-Value
Diabetes	206 (21%)	79 (18.2%)	127 (23.3%)	0.056
COPD	99 (10.1%)	37 (8.5%)	62 (11.4%)	0.147
Asthma	59 (6%)	25 (5.8%)	34 (6.2%)	0.767
IPF	13 (1.3%)	2 (0.5%)	11 (2%)	**0.035**
Dementia	138 (14.1%)	46 (10.6%)	92 (16.8%)	**0.005**
Cancer	88 (9%)	30 (6.9%)	58 (10.6%)	0.181
Cardiovascular disease	572 (58.4%)	233 (53.8%)	339 (62.1%)	**0.009**
Connective tissue disease	37 (3.8%)	10 (2.3%)	27 (4.9%)	**0.032**
Chronic kidney disease	52 (5.3%)	16 (3.7%)	36 (6.6%)	**0.045**
Chronic treatment with immunosuppressive drugs	76 (7.8%)	22 (5.1%)	54 (9.9%)	**0.005**
Charlson Comorbidity Index (median, IQR)	3 (1, 5)	3 (0, 5)	4 (1, 6)	**<0.001**

Data are presented as n (%) unless otherwise indicated. Bold indicates statistically significant differences. Abbreviations: COPD: chronic obstructive pulmonary disease; IPF: idiopathic pulmonary fibrosis.

**Table 3:** Laboratory exams [median (IQR)] and symptoms upon admission to the hospital.

Parameters	Total (n = 979)	Delta (n = 433)	Omicron (n = 546)	*p*-Value
WBC (×10^3^/μL)	6.7 (5.0, 9.5)	6.7 (4.8, 9.2)	6.8 (5.2, 9.7)	**0.047**
Lymphocytes (×10^3^/μL)	1.0 (0.7, 1.5)	1.01 (0.7, 1.38)	1.05 (0.7, 1.54)	0.114
Neutrophils (×10^3^/μL)	4.8 (3.3, 7.4)	4.7 (3.3, 7.4)	4.8 (3.3, 7.4)	0.513
Platelets (×10^3^/μL)	200 (158,260)	199 (158, 263)	200 (158, 258)	0.608
Neutrophil/lymphocyte ratio	4.4 (2.8, 8.8)	4.4 (2.9, 8.4)	4.5, (2.6, 9.0)	0.826
Platelet/lymphocyte ratio	194.2 (130.9, 289.3)	198.9 (140.2, 284.9)	188.9 (123.3, 295.9)	0.086
PCT (ng/mL)	0.07 (0.03, 0.16)	0.08 (0.05, 0.17)	0.06 (0.02,0.16)	**0.005**
Ferritin (ng/mL)	254 (136, 541)	301 (159, 644)	239 (128, 473)	**0.002**
D-dimer (mg/L)	0.73 (0.43, 1.43)	0.74 (0.44, 1.35)	0.73 (0.42, 1.51)	0.401
CRP (mg/L)	35.7 (13.3, 87.7)	40.4 (16.7, 98.5)	31.8 (11.9, 81.7)	**0.003**
LDH (U/L)	266 (211, 344)	277 (221, 375)	255 (205, 320)	**<0.001**
Symptoms				
Dyspnea N (%)	449 (45.9%)	201 (46.4%)	248 (45.4%)	0.755
Anosmia N (%)	123 (12.6%)	52 (12%)	71 (13%)	0.641
Cough N (%)	401 (41%)	186 (43%)	215 (39.4%)	0.258
Fatigue N (%)	374 (38.2%)	171 (39.5%)	203 (37.25%)	0.460
Fever N (%)	382 (39%)	193 (44.6%)	189 (34.6%)	**0.002**
Headache N (%)	171 (17.5%)	70 (16.2%)	101 (18.5%)	0.340
GI symptoms N (%)	174 (17.85)	62 (14.3%)	112 (20.5%)	**0.012**

Data are presented as median (IQR) or as n (%) unless otherwise indicated. Abbreviations: WBC: white blood cell; PCT: procalcitonin; CRP: C-reactive protein; LDH: lactate dehydrogenase test; GI: gastrointestinal symptoms.

**Table 4:** Disease severity and outcome of the study subjects during the two waves of the pandemic.

Parameters	Total (n = 979)	Delta (n = 433)	Omicron (n = 546)	*p*-Value
Length of hospital stay (days)	8 (5, 12)	8 (6, 13)	7 (5, 12)	**0.001**
PaO_2_/FiO_2_ ratio	319 (263, 366)	314 (257, 360)	322 (269, 368)	0.053
Max FiO_2_	0.32 (0.24, 0.50)	0.32 (0.24, 0.50)	0.31 (0.24, 0.50)	0.840
Admission to ICU	56 (5.7%)	28 (6.5%)	28 (5.1%)	0.37
Death	139 (14.2%)	54 (12.5%)	85 (15.6%)	0.168
Extent of disease (Co.V.A.Sc) *	**Total** **(n = 606)**	**Delta (n = 293)**	**Omicron (n = 313)**	**0.001**
1–10%	254 (41.8%)	92 (31.3%)	162 (51.8%)	
11–25%	129 (21.3%)	69 (23.5%)	60 (19.2%)	
26–50%	114 (18.8%)	71 (24.2%)	43 (13.7%)	
51–75%	71 (11.7%)	44 (15.0%)	27 (8.6%)	
>75%	38 (6.3%)	17 (5.8%)	21 (6.7%)	

* CT was available in 606 patients (293 during the period of the Delta variant predominance and 313 during the period of the Omicron predominance). Data are presented as median (IQR) or as n (%) unless otherwise indicated. Abbreviations: PaO_2_: arterial oxygen partial pressure, FiO_2_: fraction of inspired oxygen; ICU: intensive care unit; Co.V.A.Sc: COVID-19 visual assessment scale.

**Table 5:** Adverse events during hospitalization.

Events during Hospitalization	Total (n = 979)	Delta (n = 433)	Omicron (n = 546)	*p*-Value
CV event	72 (7.4%)	15 (3.5%)	57 (10.4%)	**<0.001**
PE	35 (3.6%)	13 (3%)	22 (4%)	0.387
DVT	10 (1%)	0 (0%)	10 (1.8%)	**0.005**
AKI	127 (13%)	46 (10.6%)	81 (14.8%)	0.051
ALI	91 (9.3%)	30 (6.9%)	61 (11.2%)	**0.023**

Data are presented as n (%) Abbreviations: CV: cardiovascular; PE: pneumonic embolism; DVT: deep vein thrombosis; AKI: acute kidney injury; ALI: acute liver injury.

**Table 6:** (**A**) Cox regression analysis of mortality predictors for patients hospitalized with COVID-19 during the period of the Delta variant predominance; and (**B**) Cox regression analysis of mortality predictors for patients hospitalized with COVID-19 during the period of the Omicron variant predominance.

(A)						
Variables	Univariate Analysis			Multivariate Analysis		
	HR	95% CI	*p*-Value	HR	95% CI	*p*-Value
Age	1.052	1.032–1.073	**<0.001**	1.047	1.004–1.092	**0.032**
Gender	0.961	0.564–1.638	0.883			
Obesity	0.737	0.316–1.723	0.481			
CCI	1.390	1.260–1.533	**<0.001**	1.088	0.909–1.302	0.358
Chronic treatment with immunosuppressive drugs	1.876	0.747–4.709	0.180			
N/L ratio	1.059	1.043–1.075	**<0.001**	1.046	1.015–1.078	**0.004**
D-dimer on admission	1.089	1.033–1.148	**0.001**	1.092	1.018–1.172	**0.014**
CRP on admission	1.010	1.007–1.013	**<0.001**	1.003	0.998–1.008	0.222
CV event during hospitalization	5.906	2.783–12.534	**<0.001**	3.911	1.088–14.052	**0.037**
AKI during hospitalization	8.842	5.162–15.129	**<0.001**	4.341	1.576–11.960	**0.005**
ALI during hospitalization	3.651	1.882–0.994	**<0.001**	1.071	0.308–3.722	0.914
PaO_2_/FiO_2_ on admission	0.991	0.988–0.994	**<0.001**	1.002	0.996–1.007	0.576
Extent of disease on chest CT	2.165	1.556–3.016	**<0.001**	1.902	1.194–3.030	**0.007**
**(B)**						
**Variables**	**Univariate Analysis**			**Multivariate Analysis**		
	**HR**	**95% CI**	***p*-Value**	**HR**	**95% CI**	***p*-Value**
Age	1.054	1.036–1.072	**<0.001**	1.009	0.971–1.049	0.638
Gender	0.771	0.504–1.180	0.231			
Obesity	0.324	0.131–0.801	**0.015**	0.095	0.007–1.220	0.071
CCI	1.422	1.312–1.542	**<0.001**	1.096	0.893–1.345	0.380
Chronic treatment with immunosuppressive drugs	1.379	0.732–2.597	0.320			
N/L ratio	1.053	1.039–1.067	**<0.001**	1.017	0.970–1.067	0.484
D-dimer on admission	1.069	1.043–1.097	**<0.001**	1.057	0.997–1.121	0.063
CRP on admission	1.008	1.006–1.010	**<0.001**	1.003	0.997–1.009	0.339
CV event during hospitalization	8.226	5.326–12.705	**<0.001**	3.490	1.247–9.767	**0.017**
AKI during hospitalization	10.097	6.557–15.550	**<0.001**	0.951	0.277–3.273	0.937
ALI during hospitalization	4.770	3.044–7.475	**<0.001**	4.890	1.616–14.791	**0.005**
PaO_2_/FiO_2_ on admission	0.989	0.987–0.991	**<0.001**	0.993	0.987–0.998	**0.013**
Extent of disease on chest CT	2.377	1.816–3.109	**0.001**	1.289	0.852–1.949	0.230

Abbreviations: CCI: Charlson Comorbidity Index, N/L: Neutrophils to lymphocytes ratio, CRP: C-reactive protein, CV: cardiovascular, AKI: acute kidney injury, ALI: acute liver injury, PaO_2_: arterial oxygen partial pressure, FiO_2_: fraction of inspired oxygen.

## Data Availability

Data will be available upon request.
